# Calculating Kolmogorov Complexity from the Output Frequency Distributions of Small Turing Machines

**DOI:** 10.1371/journal.pone.0096223

**Published:** 2014-05-08

**Authors:** Fernando Soler-Toscano, Hector Zenil, Jean-Paul Delahaye, Nicolas Gauvrit

**Affiliations:** 1 Grupo de Lógica, Lenguaje e Información, Universidad de Sevilla, Sevilla, Spain; 2 Unit of Computational Medicine, Karolinska Institutet, Stockholm, Sweden; 3 Laboratoire d'Informatique Fondamentale de Lille, Université de Lille I, Lille, France; 4 École Pratique des Hautes Études, Paris, France; 5 Algorithmic Nature Group, LABORES, Paris, France; UMIT, Austria

## Abstract

Drawing on various notions from theoretical computer science, we present a novel numerical approach, motivated by the notion of algorithmic probability, to the problem of approximating the Kolmogorov-Chaitin complexity of short strings. The method is an alternative to the traditional lossless compression algorithms, which it may complement, the two being serviceable for different string lengths. We provide a thorough analysis for all 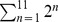 binary strings of length 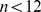 and for most strings of length 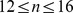 by running all 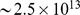 Turing machines with 5 states and 2 symbols (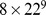 with reduction techniques) using the most standard formalism of Turing machines, used in for example the Busy Beaver problem. We address the question of stability and error estimation, the sensitivity of the continued application of the method for wider coverage and better accuracy, and provide statistical evidence suggesting robustness. As with compression algorithms, this work promises to deliver a range of applications, and to provide insight into the question of complexity calculation of finite (and short) strings.

Additional material can be found at the *Algorithmic Nature Group* website at http://www.algorithmicnature.org. An Online Algorithmic Complexity Calculator implementing this technique and making the data available to the research community is accessible at http://www.complexitycalculator.com.

## Introduction

The evaluation of the complexity of finite sequences is key in many areas of science. For example, the notions of structure, simplicity and randomness are common currency in biological systems epitomized by a sequence of fundamental nature and utmost importance: the DNA. Nevertheless, researchers have for a long time avoided any practical use of the current accepted mathematical theory of randomness, mainly because it has been considered to be useless in practice [8]. Despite this belief, related notions such as lossless uncompressibility tests have proven relative success, in areas such as sequence pattern detection [21] and have motivated distance measures and classification methods [9] in several areas (see [19] for a survey), to mention but two examples among many others of even more practical use. The method presented in this paper aims to provide sound directions to explore the feasibility and stability of the evaluation of the complexity of strings by means different to that of lossless compressibility, particularly useful for short strings. The authors known of only two similar attempts to compute the uncomputable, one related to the estimation of a Chaitin Omega number [4], and of another seminal related measure of complexity, Bennett's Logical Depth [23], [27]. This paper provides an approximation to the output frequency distribution of all Turing machines with 5 states and 2 symbols which in turn allow us to apply a central theorem in the theory of algorithmic complexity based in the notion of algorithmic probability (also known as Solomonoff's theory of inductive inference) that relates frequency of production of a string and its Kolmogorov complexity hence providing, upon application of the theorem, numerical estimations of Kolmogorov complexity by a method different to lossless compression algorithms.

A previous result [13] using a simplified version of the method reported here soon found an application in the study of economic time series [20], [34], but wider application was preempted by length and number of strings. Here we significantly extend [13] in various directions: (1) longer, and therefore a greater number–by a factor of three orders of magnitude–of strings are produced and thoroughly analyzed; (2) in light of the previous result, the new calculation allowed us to compare frequency distributions of sets from considerable different sources and of varying sizes (although the smaller is contained in the larger set, it is of negligible size in comparison) –they could have been of different type, but they are not (3) we extend the method to sets of Turing machines whose Busy Beaver has not yet been found by proposing an informed method for estimating a reasonably non-halting cutoff value based on theoretical and experimental considerations, thus (4) provide strong evidence that the estimation and scaling of the method is robust and much less dependent of Turing machine sample size, fully quantified and reported in this paper. The results reported here, the data released with this paper and the online program in the form of a calculator, have now been used in a wider number of applications ranging from psychometrics [15] to the theory of cellular automata [26], [33], graph theory and complex networks [15]. In sum this paper provides a thorough description of the method, a complete statistical analysis of the *Coding theorem method* and an online application for its use and exploitation. The calculation presented herein will remain the best possible estimation for a measure of a similar nature with the technology available to date, as an exponential increase of computing resources will improve the length and number of strings produced only linearly if the same standard formalism of Turing machines used is followed.

## Preliminaries

### Kolmogorov complexity

General and technical introductions to AIT can be found in Refs. [2], [14], [19], [32]. Central to AIT is the definition of algorithmic (Kolmogorov-Chaitin or program-size) complexity [5], [17]: 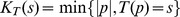
(1)where 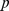 is a program that outputs 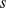 running on a universal Turing machine 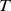. A technical inconvenience of 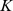 as a function taking 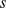 to the length of the shortest program that produces 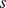 is its uncomputability. In other words, there is no program which takes a string 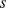 as input and produces the integer 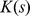 as output. This is usually considered a major problem, but one ought to expect a universal measure of complexity to have such a property. The measure was first conceived to define randomness and is today the accepted objective mathematical measure of complexity, among other reasons because it has been proven to be mathematically robust (by virtue of the fact that several independent definitions converge to it). If the shortest program 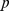 producing 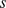 is larger than 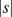, the length of 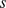, then 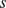 is considered random. One can approach 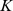 using compression algorithms that detect regularities in order to compress data. The value of the compressibility method is that the compression of a string as an approximation to 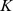 is a sufficient test of non-randomness.

It was once believed that AIT would prove useless for any real world applications [8], despite the beauty of its mathematical results (e.g. a derivation of Gödel's incompleteness theorem [6]). This was thought to be due to uncomputability and to the fact that the theory's founding theorem (the invariance theorem), left finite (short) strings unprotected against an additive constant determined by the arbitrary choice of programming language or universal Turing machine (upon which one evaluates the complexity of a string), and hence unstable and extremely sensitive to this choice.

Traditionally, the way to approach the algorithmic complexity of a string has been by using lossless compression algorithms. The result of a lossless compression algorithm is an upper bound of algorithmic complexity. However, short strings are not only difficult to compress in practice, the theory does not provide a satisfactory answer to all questions concerning them, such as the Kolmogorov complexity of a single bit (which the theory would say has maximal complexity because it cannot be further compressed). To make sense of such things and close this theoretical gap we devised an alternative methodology [13] to compressibility for approximating the complexity of short strings, hence a methodology applicable in many areas where short strings are often investigated (e.g. in bioinformatics). This method has yet to be extended and fully deployed in real applications, and here we take a major step towards full implementation, providing details of the method as well as a thorough theoretical analysis.

### Invariance and compression

A fair compression algorithm is one that transforms a string into two components. The first of these is the compressed version while the other is the set of instructions for decompressing the string. Both together account for the final length of the compressed version. Thus the compressed string comes with its own decompression instructions. Paradoxically, lossless compression algorithms are more stable the longer the string. In fact the invariance theorem guarantees that complexity values will only diverge by a constant 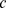 (e.g. the length of a compiler, a translation program between 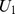 and 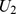) and will converge at the limit.

#### Invariance Theorem [2], [19]

If 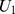 and 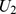 are two universal Turing machines and 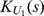 and 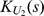 the algorithmic complexity of 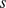 for 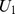 and 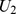, there exists a constant 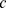 such that: 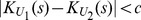
(2)


Hence the longer the string, the less important the constant 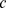 or choice of programming language or universal Turing machine. However, in practice 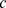 can be arbitrarily large, thus having a very great impact on finite short strings. Indeed, the use of data lossless compression algorithms as a method for approximating the Kolmogorov complexity of a string is accurate in direct proportion to the length of the string.

### Solomonoff-Levin Algorithmic Probability

The algorithmic probability (also known as Levin's semi-measure) of a string 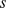 is a measure that describes the expected probability of a random program 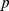 running on a universal *prefix-free* Turing machine 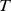 producing 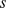. The group of valid programs forms a prefix-free set, that is no element is a prefix of any other, a property necessary for 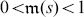 (for details see [2]).Formally [5], [18], [24], 
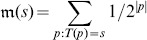
(3)


Levin's semi-measure 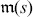 defines the so-called Universal Distribution [16]. Here we propose to use 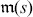 as an alternative to the traditional use of compression algorithms to calculate 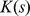 by means of the following theorem.

#### Coding Theorem [18]

There exists a constant 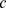 such that: 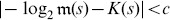
(4)


That is, if a string has many long descriptions it also has a short one [10]. It beautifully connects frequency to complexity–the frequency (or probability) of occurrence of a string with its algorithmic (Kolmogorov) complexity. It is called a *semi* measure because the sum is not always 1, unlike probability measures. This is due to the Turing machines that never halt. The coding theorem implies that [13] one can calculate the Kolmogorov complexity of a string from its frequency [11]–[13], [29], simply rewriting the formula as: 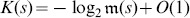
(5)


An important property of 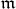 as a semi-measure is that it dominates any other effective semi-measure 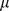 because there is a constant 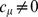 such that for all 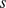, 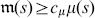 hence called *Universal* [16].

### The Busy Beaver function

#### Notation

We denote by 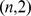 the class (or space) of all 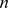-state 2-symbol Turing machines (with the halting state not included among the 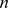 states).

In addressing the problem of approaching 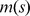 by running computer programs (in this case deterministic Turing machines) one can use the known values of the so-called Busy Beaver functions as suggested by and used in [13], [29]. The Busy Beaver functions 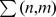 and 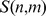 can be defined as follows:

#### Busy Beaver functions (Rado [22])

If 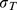 is the number of ‘1s’ on the tape of a Turing machine 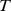 with 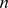 states and 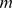 symbols upon halting starting from a blank tape (no input), then the Busy Beaver function 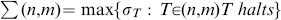. Alternatively, if 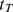 is the number of steps that a machine 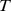 takes before halting from a blank tape, then 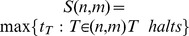.

In other words, the Busy Beaver functions are the functions that return the longest written tape and longest runtime in a set of Turing machines with 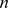 states and 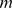 symbols. 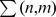 and 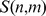 are noncomputable functions by reduction to the halting problem. In fact 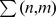 grows faster than any computable function can. Nevertheless, exact values can be calculated for small 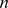 and 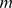, and they are known for, among others, 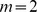 symbols and 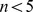 states. A program showing the evolution of all known Busy Beaver machines developed by one of this paper's authors is available online [31].

**Figure 1:**
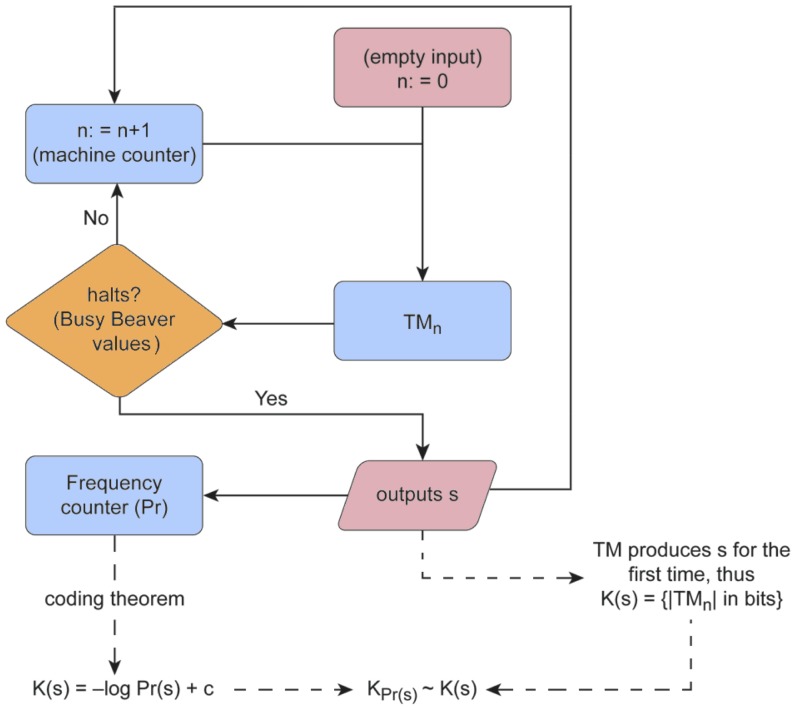
A flow chart illustrating the *Coding Theorem Method*, a never-ending algorithm for evaluating the (Kolmogorov) complexity of a (short) string making use of several concepts and results from theoretical computer science, in particular the halting probability, the Busy Beaver problem, Levin's semi-measure and the Coding theorem. The Busy Beaver values can be used up to 4 states for which they are known, for more than 4 states an informed maximum runtime is used as described in this paper, informed by theoretical [3] and experimental (Busy Beaver values) results. Notice that 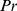 are the probability values calculated dynamically by running an increasing number of Turing machines. 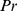 is intended to be an approximation to 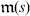 out of which we build 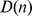 after application of the Coding theorem.

This allows one to circumvent the problem of noncomputability for small Turing machines of the size that produce short strings whose complexity is approximated by applying the algorithmic Coding theorem (see Fig. 1). As is widely known, the Halting problem for Turing machines is the problem of deciding whether an arbitrary Turing machine 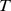 eventually halts on an arbitrary input 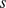. Halting computations can be recognized by running them for the time they take to halt. The problem is to detect non-halting programs, programs about which one cannot know in advance whether they will run forever or eventually halt.

### The Turing machine formalism

It is important to describe the Turing machine formalism because numerical values of algorithmic probability for short strings will be provided under this chosen standard model of a Turing machine.

Consider a Turing machine with the binary alphabet 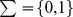 and 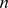 states 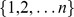 and an additional Halt state denoted by 0 (as defined by Rado in his original Busy Beaver paper [22]).

The machine runs on a 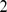-way unbounded tape. At each step:

1. the machine's current “state”; and
2. the tape symbol the machine's head is scanning

define each of the following:

1. a unique symbol to write (the machine can overwrite a 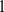 on a 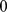, a 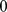 on a 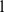, a 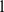 on a 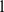, and a 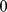 on a 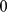);
2. a direction to move in: 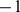 (left), 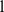 (right) or 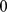 (none, when halting); and
3. a state to transition into (which may be the same as the one it was in).

The machine halts if and when it reaches the special halt state 0. There are 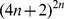 Turing machines with 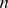 states and 2 symbols according to the formalism described above. The output string is taken from the number of contiguous cells on the tape the head of the halting 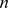-state machine has gone through. A machine produces a string upon halting.

## Methodology

One can attempt to approximate 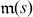 (see Eq. 3) by running every Turing machine an particular enumeration, for example, a quasi-lexicographical ordering, from shorter to longer (with number of states 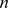 and 2 fixed symbols). It is clear that in this fashion once a machine produces 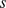 for the first time, one can directly calculate an approximation of 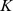, because this is the length of the first Turing machine in the enumeration of programs of increasing size that produces 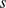. But more important, one can apply the Coding theorem to extract 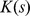 from 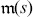 directly from the output distribution of halting Turing machines. Let's formalize this by using the function 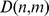 as the function that assigns to every string 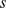 produced in 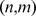 the quotient: (number of times that a machine in 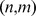 produces 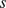)/(number of machines that halt in 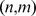) as defined in [13], [29]. More formally, 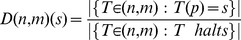
(6)


Where 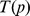 is the Turing machine with number 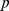 (and empty input) that produces 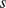 upon halting and 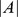 is, in this case, the cardinality of the set 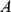. A variation of this formula closer to the definition of 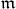 is given by: 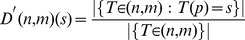
(7)



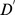
 is strictly smaller than 1 for 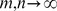, because of the Turing machines that never halt, just as it occurs for 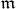. However, for fixed 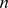 and 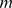 the sum of 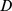 will always be 1. We will use Eq. 6 for practical reasons, because it makes the frequency values more readable (most machines don't halt, so those halting would have a tiny fraction with too many leading zeros after the decimal). Moreover, the function 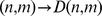 is non-computable [13], [29] but it can be approximated from below, for example, by running small Turing machines for which known values of the Busy Beaver problem [22] are known. For example [1], for 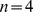, the Busy Beaver function for maximum runtime 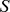, tells us that 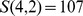, so we know that a machine running on a blank tape will never halt if it hasn't halted after 107 steps, and so we can stop it manually. In what follows we describe the exact methodology. From now on, 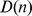 with a single parameter will mean 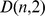. We call this method the *Coding Theorem Method* to approximate 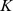 (which we will denote by 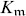).

### Kolmogorov complexity from the output frequency of small Turing machines

Approximations from the output distribution of Turing machines with 2 symbols and 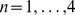 states for which the Busy Beaver values are known were estimated before [13], [29] but for the same reason the method was not scalable beyond 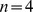. The formula for the number of machines given a number of states 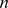 is given by 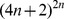 derived from the formalism described. There are 26 559 922 791 424 Turing machines with 5 states (that is, for the reader amusement, about the same number of red cells in the blood of an average adult). Here we describe how an optimal runtime based on theoretical and experimental grounds can be calculated to scale the method to larger sets of small Turing machines.

Because there are a large enough number of machines to run even for a small number of machine states (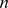), applying the Coding theorem provides a finer and increasingly stable evaluation of 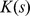 based on the frequency of production of a large number of Turing machines, but the number of Turing machines grows exponentially, and producing 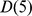 requires considerable computational resources.

### Setting an informed runtime

The Busy Beaver for Turing machines with 4 states is known to be 107 steps [1], that is, any Turing machine with 2 symbols and 4 states running longer than 107 steps will never halt. However, the exact number is not known for Turing machines with 2 symbols and 5 states, although it is believed to be 47 176 870, as there is a candidate machine that runs for this long and halts and no machine greater runtime has yet been found.

So we decided to let the machines with 5 states run for 4.6 times the Busy Beaver value for 4-state Turing machines (for 107 steps), knowing that this would constitute a sample significant enough to capture the behavior of Turing machines with 5 states. The chosen runtime was rounded to 500 steps, which was used to build the output frequency distribution for 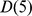. The theoretical justification for the pertinence and significance of the chosen runtime is provided in the following sections.

### Reduction techniques

We didn't run all the Turing machines with 5 states to produce 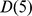 because one can take advantage of symmetries and anticipate some of the behavior of the Turing machines directly from their transition tables without actually running them (this is impossible in general due to the halting problem). We avoided some trivial machines whose results we know without having to run them (reduced enumeration). Also, some non-halting machines were detected before consuming all the runtime (filters). Table 1 shows the reductions utilized for the number of total machines in order to decrease the computing time for the approximation of 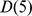.

**Table 1:** Non-halting machines filtered.

Filter	number of TMs
machines without transitions to the halting state	1 610 612 736
short escapees	464 009 712
other escapees	336 027 900
cycles of period two	15 413 112
machines that consume all the runtime	366 784 524
**Total**	2792847984

### Exploiting symmetries

#### Symmetry of 0 and 1

The blank symbol is one of the 2 symbols (0 or 1) in the first run, while the other is used in the second run (in order to avoid any asymmetries due to the choice of a single blank symbol). In other words, we considered two runs for each Turing machine, one with 0 as the blank symbol (the symbol with which the tape starts out and fills up), and an additional run with 1 as the blank symbol. This means that every machine was run twice. Due to the symmetry of the computation, there is no real need to run each machine twice; one can *complete* the string frequencies by assuming that each string produced produced by a Turing machine has its complement produced by another symmetric machine with the same frequency, we then group and divide by symmetric groups. We used this technique from 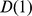 to 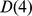. A more detailed explanation of how this is done is provided in [13], [29] using Polya's counting theorem.

#### Symmetry right-left

We can exploit the right-left symmetry. We may, for example, run only those machines with an initial transition (initial state and blank symbol) moving to the right and to a state different from the initial one (because an initial transition to the initial state produces a non-halting machine) and the halting one (these machines stop in just one step and produce ‘0’ or ‘1’).

For every string produced, we also count the reverse in the tables. We count the corresponding number of one-symbol strings and non-halting machines as well.

#### Reduction techniques by exploiting symmetries

If we consider only machines with a starting transition that moves to the right and goes to a state other than the starting and halting states, the number of machines is given by 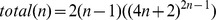



Note that for the starting transition there are 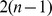 possibilities (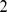 possible symbols to write and 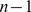 possible new states, as we exclude the starting and halting states). For the other 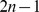 transitions there are 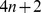 possibilities.

We can make an enumeration from 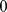 to 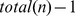. Of course, this enumeration is not the same as the one we use to explore the whole space. The same number will not correspond to the same machine.

In the whole 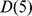 space there are 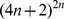 machines, so it is a considerable reduction. This reduction in 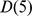 means that in the reduced enumeration we have 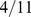 of the machines we had in the original enumeration.

### Strings to complete after running the reduced enumeration

Suppose that using the previous enumeration we run 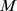 machines for 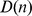 with blank symbol 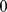. 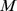 can be the total number of machines in the reduced space or a random number of machines in it (such as we use to study the runtime distribution, as it is better described below).

For the starting transition we considered only 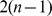 possibilities out of 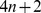 possible transitions in the whole space. Then, we proceeded as follows to complete the strings produced by the 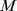 runs.

1. We avoided 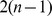 transitions moving left to a different state than the halting and starting ones. We completed such transitions by reversing all the strings found. Non-halting machines were multiplied by 2.
2. We also avoided 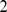 transitions (writing ‘0’ or ‘1’) from the initial to the halting state. We completed such transitions by
  - Including 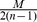 times ‘0’.
  - Including 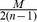 times ‘1’.
3. Finally, we avoided 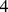 transitions from the initial state to itself (2 movements ×2 symbols). We completed by including 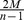 non-halting machines.

With these completions, we obtained the output strings for the blank symbol 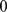. To complete for the blank symbol 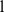 we took the complement to 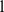 of each string produced and counted the non-halting machines twice.

Then, by running 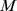 machines, we obtained a result representing 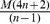, that for 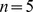 is 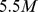.

### Detecting non-halting machines

It is useful to avoid running machines that we can easily check that will not stop. These machines will consume the runtime without yielding an output.

The reduction in the enumeration that we have shown reduces the number of machines to be generated. Now we present some reductions that work after the machines are generated, in order to detect non-halting computations and skip running them. Some of these were detected when filling the transition table, others at runtime.

#### Machines without transitions to the halting state

While we are filling the transition table, if a certain transition goes to the halting state, we can activate a flag. If after completing the transition table the flag is not activated, we know that the machine won't stop.

In our reduced enumeration there are 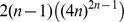 machines of this kind. In 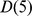 this is 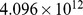 machines. It represents 42.41% of the total number of machines.

The number of machines in the reduced enumeration that are not filtered as non-halting when filling the transition table is 5 562 153 742 336. That is 504.73 times the total number of machines that fully produce 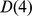.

#### Detecting escapees

There should be a great number of escapees, that is, machines that run infinitely in the same direction over the tape.

Some kinds are simple to check in the simulator. We can use a counter that indicates the number of consecutive not-previously-visited tape positions that the machines visits. If the counter exceeds the number of states, then we have found a loop that will repeat infinitely. To justify this, let us ask you to suppose that at some stage the machine is visiting a certain tape-position for the first time, moving in a specific direction (the direction that points toward new cells). If the machine continues moving in the same direction for 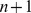 steps, and thus reading blank symbols, then it has repeated some state 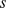 in two transitions. As it is always reading (not previously visited) blank symbols, the machine has repeated the transition for 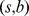 twice, 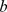 being the blank symbol. But the behavior is deterministic, so if the machine has used the transition for 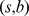 and after some steps in the same direction visiting blank cells, it has repeated the same transition, it will continue doing so forever, because it will always find the same symbols.

There is another possible direction in which this filter may apply: if the symbol read is a blank one not previously visited, the shift is in the direction of new cells and there is no modification of state. In fact this would be deemed an escapee, because the machine runs for 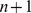 new positions over the tape. But it is an escapee that is especially simple to detect, in just one step and not 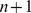. We call the machines detected by this simple filter “short escapees”, to distinguish them from other, more general escapees.

#### Detecting cycles

We can detect cycles of period two. They are produced when in steps 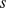 and 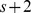 the tape is identical and the machine is in the same state and the same position. When this is the case, the cycle will be repeated infinitely. To detect it, we have to anticipate the following transition that will apply in some cases. In a cycle of period two, the machine cannot change any symbol on the tape, because if it did, the tape would be different after two steps. Then the filter would be activated when there is a transition that does not change the tape, for instance 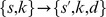
where 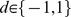 is some direction (left, right) and the head is at position 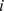 on tape 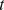, which is to say, reading the symbol 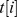. Then, there is a cycle of period two if and only if the transition that corresponds to 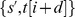 is 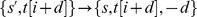



### Number of Turing machines

We calculated 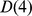 with and without all the filters as suggested in [13]. Running 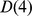 without reducing the number or detecting non-halting machines took 952 minutes. Running the reduced enumeration with non-halting detectors took 226 minutes.

Running all the Turing machines with 5 states in the reduced enumeration up to 500 steps for the calculation of 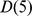 took 18 days using 25×86−64 CPUs running at 2128 MHz with 4 GB of memory each (a supercomputer located at the Centro Informático Científico de Andalucía (CICA), Spain). In order to save space in the output of 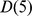, our C++ simulator produced partial results every 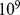 consecutive machines according to the enumeration. Every 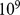 machines, the counters for each string produced were updated. The final classification is only 4.1 Megabytes but we can estimate the size of the output had we not produced partial results on the order of 1.28 Terabytes for the reduced space and 6.23 Terabytes for the full one. If we were to include in the output an indication for non-halting machines, the files would grow an extra 1.69 Terabytes for the reduced enumeration and 8.94 Terabytes for the full one.

## Results

Samples of strings extracted from the output frequency of these machines are shown in Tables 2, 3, 4, 5, 6, 7 and 8; highlighting various important features found in the distributions. Table 2 provides a glance at 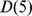 showing the 147 most frequent (and therefore simplest) calculated strings out of 99 608. The top strings of 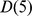 conform to an intuition of simplicity. Table 3 shows all the 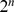 strings for 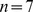, hence displaying what 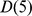 suggests are the strings sorted from lowest to highest complexity, which seems to agree well with the intuition of simple (from top left) to random-looking (bottom right).

**Table 2:** The 147 most frequent strings from *D*(5) (by row).

1	1	0	11	10	01	00	111
8	000	110	100	011	001	101	010
15	1111	0000	1110	1000	0111	0001	1101
22	1011	0100	0010	1010	0101	1100	0011
29	1001	0110	11111	00000	11110	10000	01111
36	00001	11101	10111	01000	00010	11011	00100
43	10110	10010	01101	01001	10101	01010	11010
50	10100	01011	00101	11100	11000	00111	00011
57	11001	10011	01100	00110	10001	01110	111111
64	000000	111110	100000	011111	000001	111101	101111
71	010000	000010	101010	010101	101101	010010	111011
78	110111	001000	000100	110101	101011	010100	001010
85	101001	100101	011010	010110	110110	100100	011011
92	001001	111100	110000	001111	000011	101110	100010
99	011101	010001	110010	101100	010011	001101	111001
106	100111	011000	000110	111010	101000	010111	000101
113	100110	011001	110011	001100	100001	011110	110100
120	001011	111000	000111	110001	100011	011100	001110
127	1111111	0000000	1111110	1000000	0111111	0000001	1010101
134	0101010	1111101	1011111	0100000	0000010	1111011	1101111
141	0010000	0000100	1110111	0001000	1111100	1100000	0011111

The first column is a counter to help locate the rank of each string.

**Table 3:** All the 2*^n^* strings for *n* = 7 from *D*(5) sorted from highest frequency (hence lowest complexity) to lowest frequency (hence highest (random) complexity).

1	1111111	0000000						
2	1111110	1000000	0111111	0000001				
3	1010101	0101010						
4	1111101	1011111	0100000	0000010				
5	1111011	1101111	0010000	0000100				
6	1110111	0001000						
7	1111100	1100000	0011111	0000011				
8	1011010	1010010	0101101	0100101				
9	1101101	1011011	0100100	0010010	1111001	1001111	0110000	0000110
10	1110101	1010111	0101000	0001010				
11	1101110	1000100	0111011	0010001				
12	1101010	1010100	0101011	0010101				
13	1010110	1001010	0110101	0101001				
14	1111010	1010000	0101111	0000101				
15	1110110	1001000	0110111	0001001				
16	1010001	1000101	0111010	0101110				
17	1011110	1000010	0111101	0100001				
18	1011101	0100010						
19	1101011	0010100	1001001	0110110				
20	1110011	1100111	0011000	0001100				
21	1100101	1010011	0101100	0011010				
22	1011001	1001101	0110010	0100110	1000001	0111110		
23	1111000	1110000	0001111	0000111	1101001	1001011	0110100	0010110
24	1110010	1011000	0100111	0001101	1101100	1100100	0011011	0010011
25	1100010	1011100	0100011	0011101				
26	1100110	1001100	0110011	0011001				
27	1001110	1000110	0111001	0110001				
28	1100001	1000011	0111100	0011110				
29	1110001	1000111	0111000	0001110				
30	1100011	0011100						
31	1110100	1101000	0010111	0001011				

Strings in each row have the same frequency (hence the same Kolmogorov complexity). There are 31 different groups representing the different complexities of the 2^7^ = 128 strings.

**Table 4:** Minimal examples of emergence: the first 50 climbers.

00000000	000000000	000000001	000010000	010101010
000000010	000000100	0000000000	0101010101	0000001010
0010101010	00000000000	0000000010	0000011010	0100010001
0000001000	0000101010	01010101010	0000000011	0101010110
0000000100	0000010101	000000000000	0000110000	0000110101
0000000110	0110110110	00000010000	0000001001	00000000001
0010101101	0101001001	0000011000	00010101010	01010010101
0010000001	00000100000	00101010101	00000000010	00000110000
00000000100	01000101010	01010101001	01001001001	010101010101
01001010010	000000000001	00000011000	00000000101	0000000000000

**Table 5:** The 20 strings for which 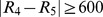.

sequence	*R*_4_	*R*_5_
010111110	1625.5	837.5
011111010	1625.5	837.5
100000101	1625.5	837.5
101000001	1625.5	837.5
000011001	1625.5	889.5
011001111	1625.5	889.5
100110000	1625.5	889.5
111100110	1625.5	889.5
001111101	1625.5	963.5
010000011	1625.5	963.5
101111100	1625.5	963.5
110000010	1625.5	963.5
0101010110	1625.5	1001.5
0110101010	1625.5	1001.5
1001010101	1625.5	1001.5
1010101001	1625.5	1001.5
0000000100	1625.5	1013.5
0010000000	1625.5	1013.5
1101111111	1625.5	1013.5
1111111011	1625.5	1013.5

**Table 6:** Top 20 strings in *D*(5) with highest frequency and therefore lowest Kolmogorov (program-size) complexity.

sequence	frequency ( 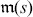 )	complexity ( 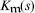 )
1	0.175036	2.51428
0	0.175036	2.51428
11	0.0996187	3.32744
10	0.0996187	3.32744
01	0.0996187	3.32744
00	0.0996187	3.32744
111	0.0237456	5.3962
000	0.0237456	5.3962
110	0.0229434	5.44578
100	0.0229434	5.44578
011	0.0229434	5.44578
001	0.0229434	5.44578
101	0.0220148	5.50538
010	0.0220148	5.50538
1111	0.0040981	7.93083
0000	0.0040981	7.93083
1110	0.00343136	8.187
1000	0.00343136	8.187
0111	0.00343136	8.187
0001	0.00343136	8.187

From frequency (middle column) to complexity (extreme right column) applying the coding theorem in order to get 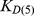 which we will call 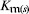 as our current best approximation to an experimental 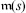, that is 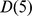, through the Coding theorem.

**Table 7:** 20 random strings (sorted from lowest to highest complexity values) from the first half of *D*(5) to which the coding theorem has been applied (extreme right column) to approximate *K*(*s*).

string length	string	complexity ( 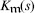 )
11	11011011010	28.1839
12	101101110011	32.1101
12	110101001000	32.1816
13	0101010000010	32.8155
14	11111111100010	34.1572
12	011100100011	34.6045
15	001000010101010	35.2569
16	0101100000000000	35.6047
13	0110011101101	35.8943
15	101011000100010	35.8943
16	1111101010111111	25.1313
18	000000000101000000	36.2568
15	001010010000000	36.7423
15	101011000001100	36.7423
17	10010011010010011	37.0641
21	100110000000110111011	37.0641
14	11000010000101	37.0641
17	01010000101101101	37.4792
29	01011101111100011101111010101	37.4792
14	11111110011110	37.4792

**Table 8:** Bottom 21 strings of length *n* = 12 with smallest frequency in *D*(5).

100111000110	100101110001	100011101001
100011100001	100001110001	011110001110
011100011110	011100010110	011010001110
011000111001	000100110111	111000110100
110100111000	001011000111	000111001011
110100011100	110001110100	001110001011
001011100011	110000111100	001111000011

### Reliability of the approximation of 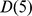


**Figure 2:**
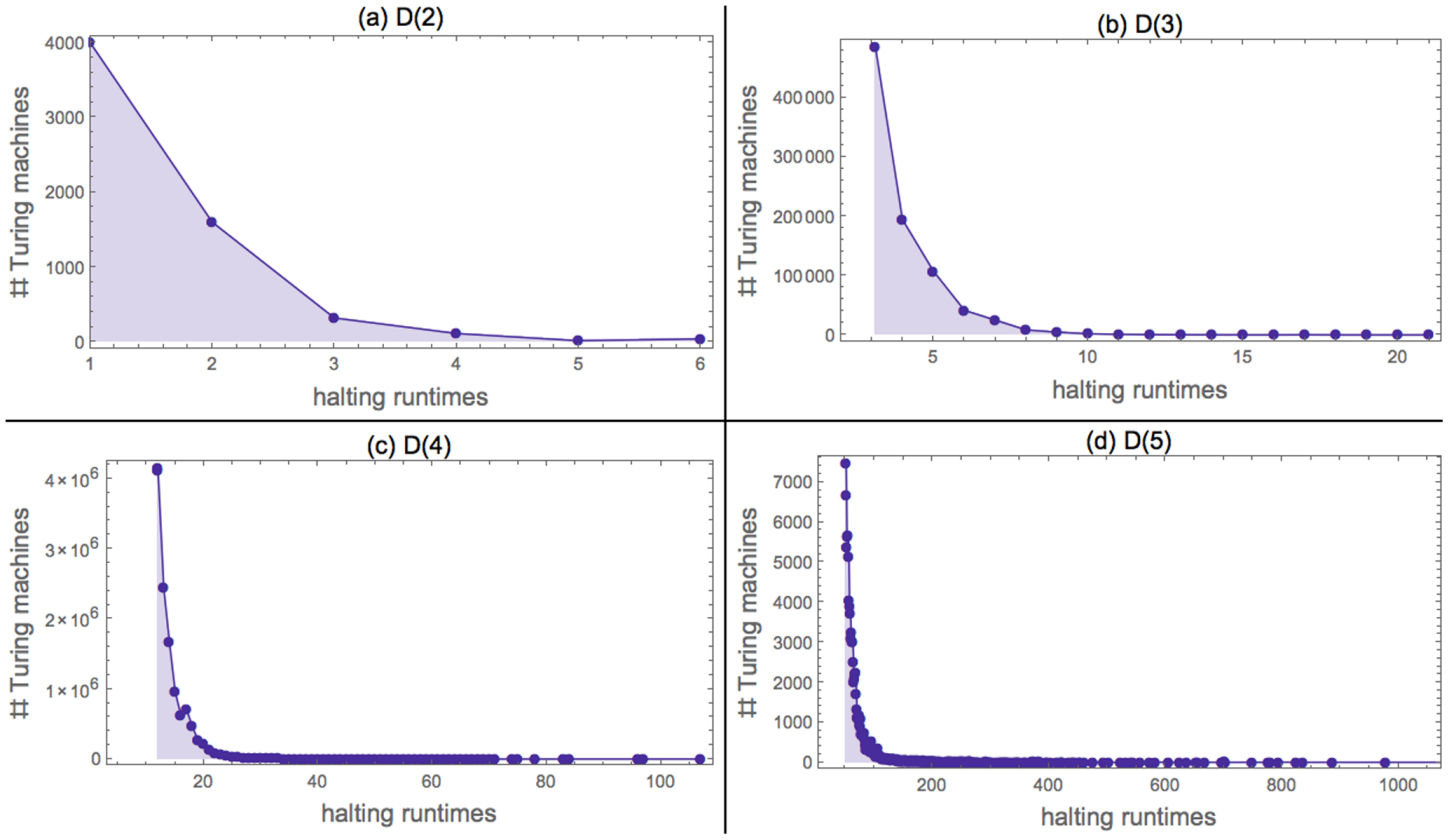
Distribution of runtimes from *D*(2) to *D*(5). On the *y*-axes are the number of Turing machines and on the *x*-axes the number of steps upon halting. For 5-state Turing machines no Busy Beaver values are known, hence *D*(5) (Fig. d) was produced by Turing machines with 5 states that ran for at most 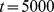 steps. These plots show, however, that the runtime cutoff 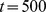 for the production of *D*(5) covers most of the halting Turing machines when taking a sample of 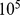 machines letting them run for up to 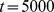 steps, hence the missed machines in *D*(5) must be a negligible number for 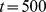.

Not all 5-state Turing machines have been used to build 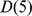, since only the output of machines that halted at or before 500 steps were taken into consideration. As an experiment to see how many machines we were leaving out, we ran 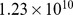 Turing machines for up to 5000 steps (see Fig. 2d). Among these, only 50 machines halted after 500 steps and before 5000 (that is less than 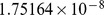 because in the reduced enumeration we don't include those machines that halt in one step or that we know won't halt before generating them, so it is a smaller fraction), with the remaining 1 496 491 379 machines not halting at 5000 steps. As far as these are concerned–and given the unknown values for the Busy Beavers for 5 states–we do not know after how many steps they would eventually halt, if they ever do. According to the following analysis, our election of a runtime of 500 steps therefore provides a good estimation of 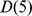.

The frequency of runtimes of (halting) Turing machines has theoretically been proven to drop exponentially [3], and our experiments are closer to the theoretical behavior (see Fig. 2). To estimate the fraction of halting machines that were missed because Turing machines with 5 states were stopped after 500 steps, we hypothesize that the number of steps 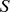 a random halting machine needs before halting is an exponential RV (random variable), defined by 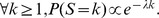 We do not have direct access to an evaluation of 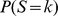, since we only have data for those machines for which 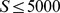. But we may compute an approximation of 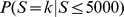, 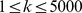, which is proportional to the desired distribution.

**Figure 3:**
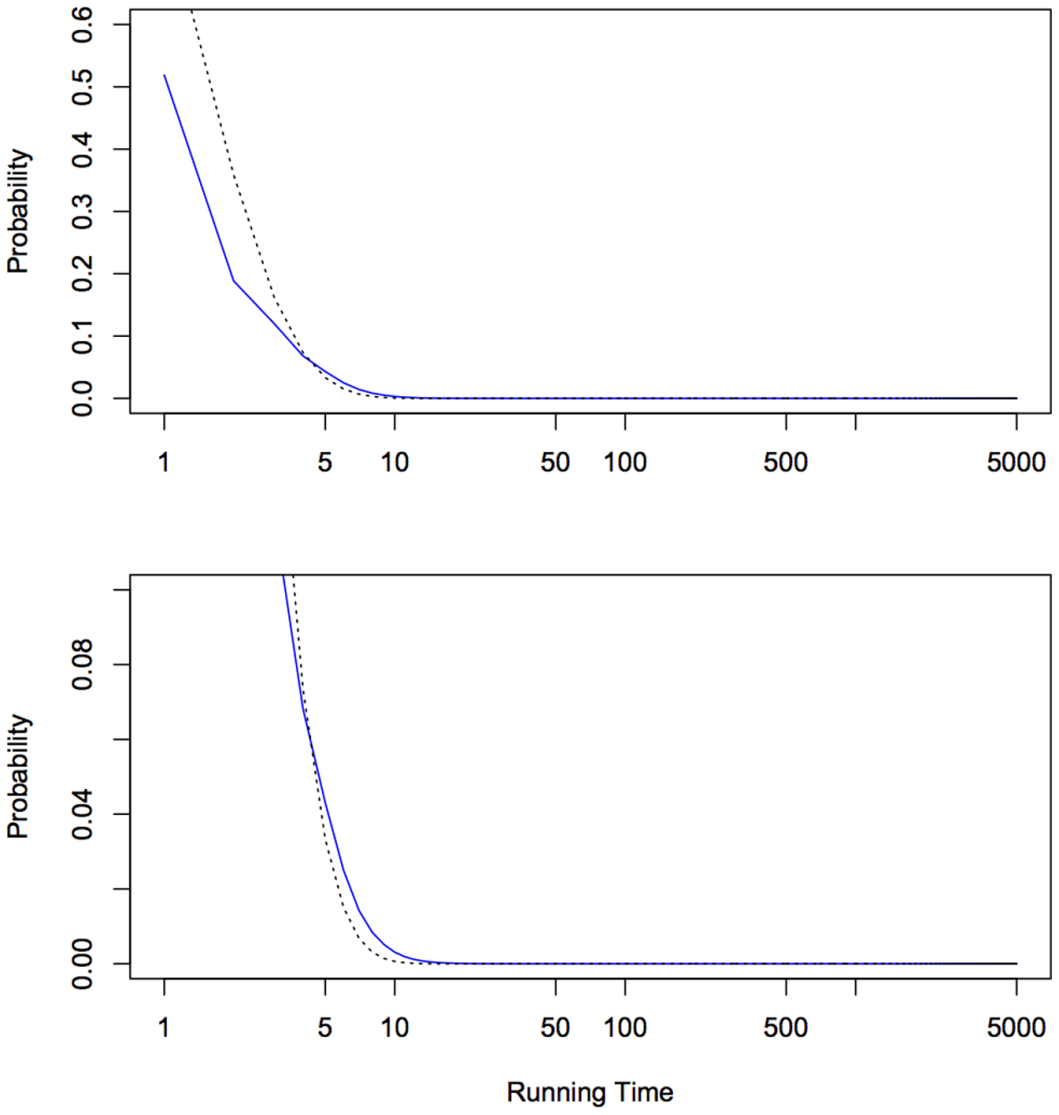
Observed (solid) and theoretical (dotted) 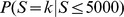 against *k*. The *x*-axis is logarithmic. Two different scales are used on the *y*-axis to allow for a more precise visualization.

A non-linear regression using ordinary least-squares gives the approximation 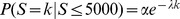 with 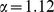 and 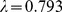. The residual sum-of-squares is 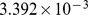, the number of iterations 9 with starting values 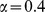 and 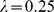. Fig. 3 helps to visualize how the model fits the data.

The model's 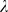 is the same 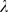 appearing in the general law 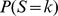, and may be used to estimate the number of machines we lose by using a 500 step cut-off point for running time: 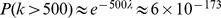. This estimate is far below the point where it could seriously impair our results: the less probable (non-impossible) string according to 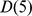 has an observed probability of 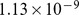.

Although this is only an estimate, it suggests that missed machines are few enough to be considered negligible.

### Features of *D*(5)

#### Lengths

**Figure 4:**
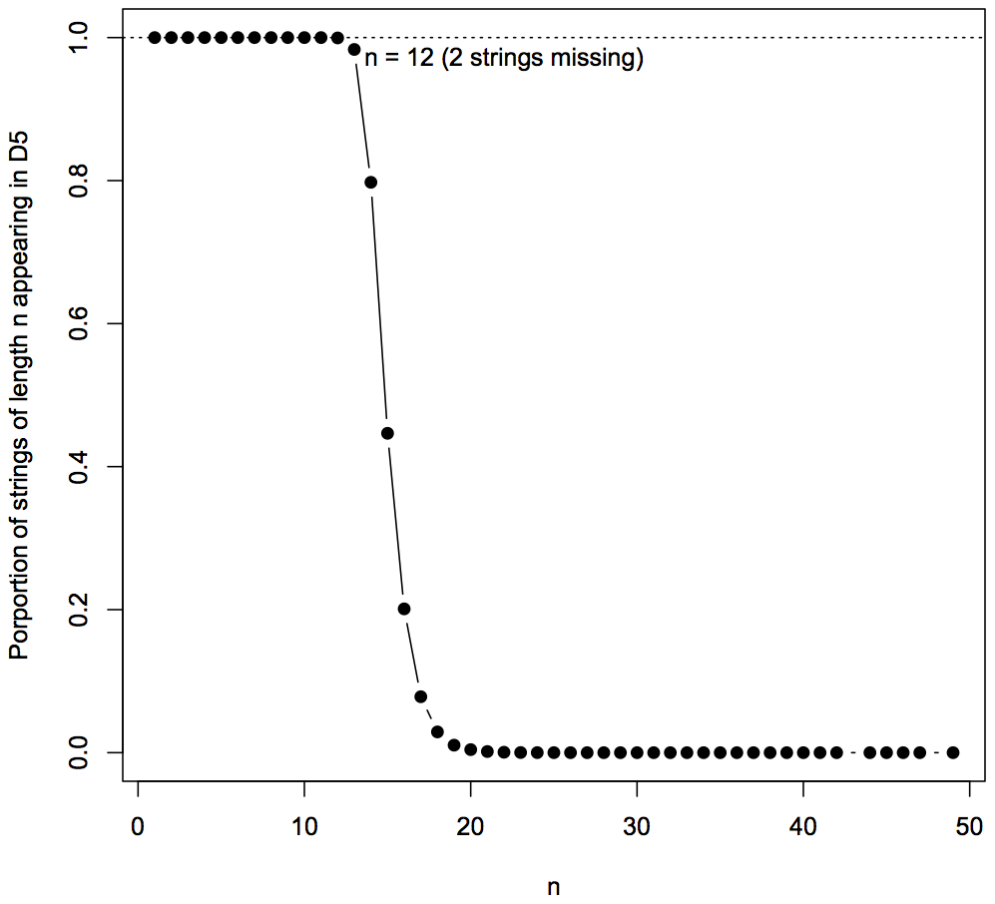
Proportion of all *n*-long strings appearing in *D*(5) against *n*.

5-state Turing machines produced 99 608 different binary strings (to be compared to the 1832 strings for 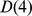). While the largest string produced for 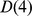 was of length 16 bits and only all 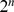 strings for 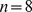 were produced, the strings in 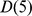 have lengths from 1 to 49 bits (excluding lengths 42 and 46 that never occur) and include every possible string of length 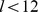. Among the 12 bit strings, only two were not produced (000110100111 and 111001011000). Of 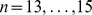 about half the 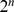 strings were produced (and therefore have frequency and complexity values). Fig. 4 shows the proportion of 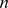-long strings appearing in 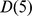 outputs, for 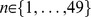.

**Figure 5:**
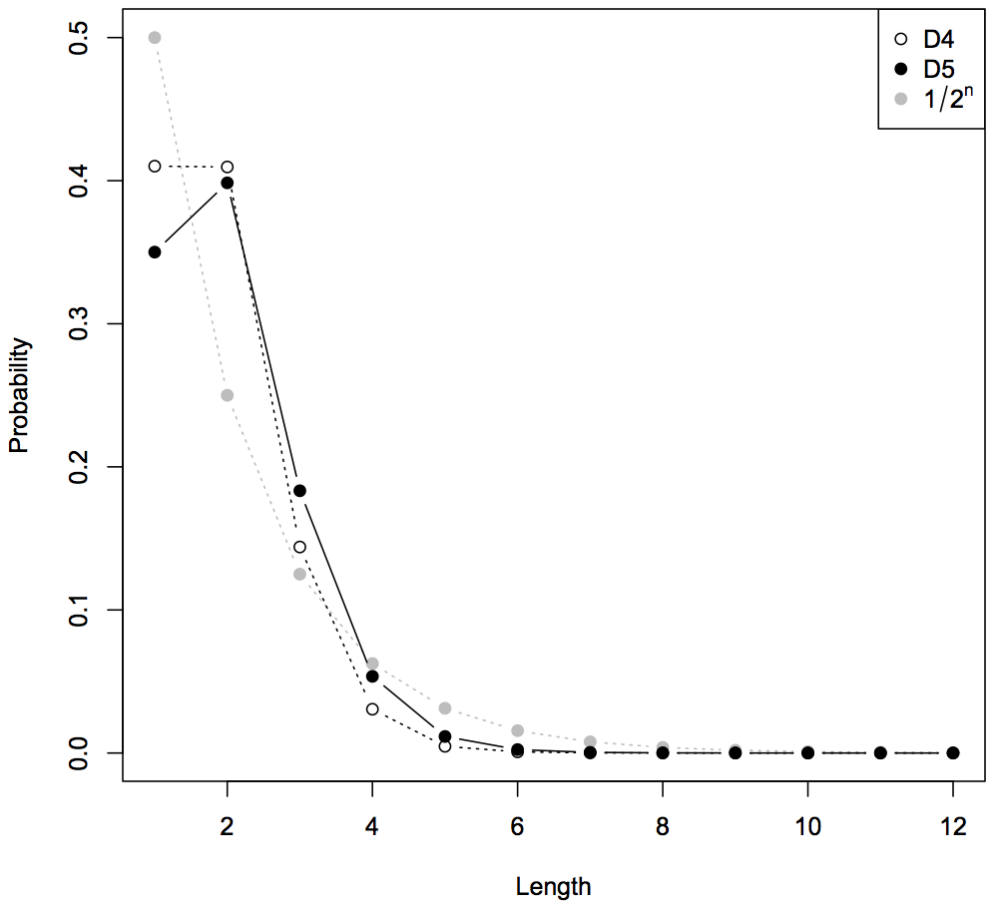
Cumulative probability of all *n*-long strings against *n*.

The cumulative probability of every 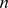-long string gives a probability law on 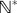. Fig. 5 shows such a law obtained with 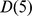, with 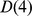, and with the theoretical 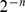 appearing in Levin's semi-measure. The most important difference may be the fact that this law does not decrease for 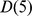, since length 2 is more likely than length 1.

#### Global simplicity

Some binary sequences may seem simple from a global point of view because they show symmetry (1011 1101) or repetition (1011 1011). Let us consider the string 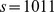 as an example. We have 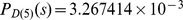. The repetition 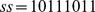 has a much lower probability 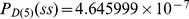. This is not surprising considering the fact that 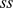 is much longer than 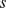, but we may then wish to consider other strings based on 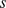. In what follows, we will consider three methods (repetition, symmetrization, 0-complementation). The repetition of 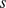 is 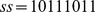, the “symmetrized” 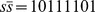, and the 0-complementation 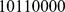. These three strings of identical length have different probabilities (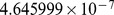, 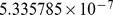 and 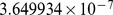 respectively).

**Figure 6:**
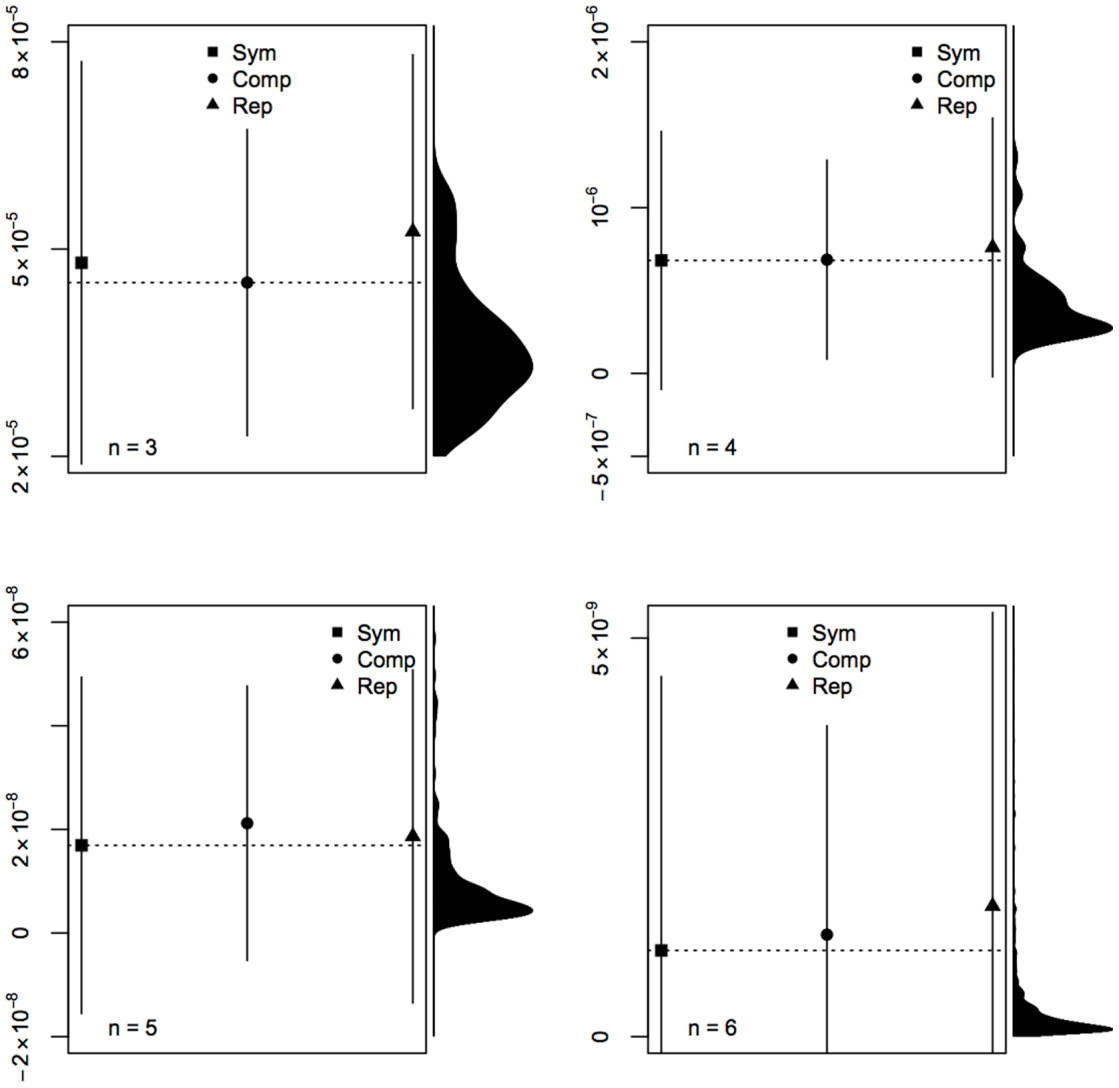
Mean ± standard deviation of *D*(5) of 2*n*-long strings given by process of symmetrization (Sym), 0-complementation (Comp) and repetition (Rep) of all *n*-long strings. The dotted horizontal line shows the minimum mean among Sym, Comp and Rep. The density of *D*(5) (smoothed with Gaussian kernel) for all 2*n*-long strings is given in the right-margin.

Let us now consider all strings of length 3 to 6, and their symmetrization, 0-complementation and repetition. Fig. 6 is a visual presentation of the results. In each case, even the minimum mean between the mean of symmetrized, complemented and repeated patterns (dotted horizontal line) lies in the upper tail of the 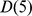 distribution for 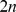-length strings. And this is even more obvious with longer strings. Symmetry, complementation and repetition are, on average, recognized by 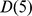.

Another method for finding “simple” sequences is based on the fact that the length of a string is negatively linked to its probability of appearing in 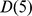. When ordered by decreasing probability, strings show increasing lengths. Let's call those sequences for which length is greater than that of the next string “climbers”. The first 50 climbers appearing in 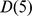 are given in Table 4 and show subjectively simple patterns, as expected.

Strings are not sorted by length but follow an interesting distribution of length differences that agrees with our intuition of simplicity and randomness and is in keeping with the expectation from an approximation to 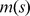 and therefore 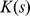.

#### Binomial behavior

**Figure 7:**
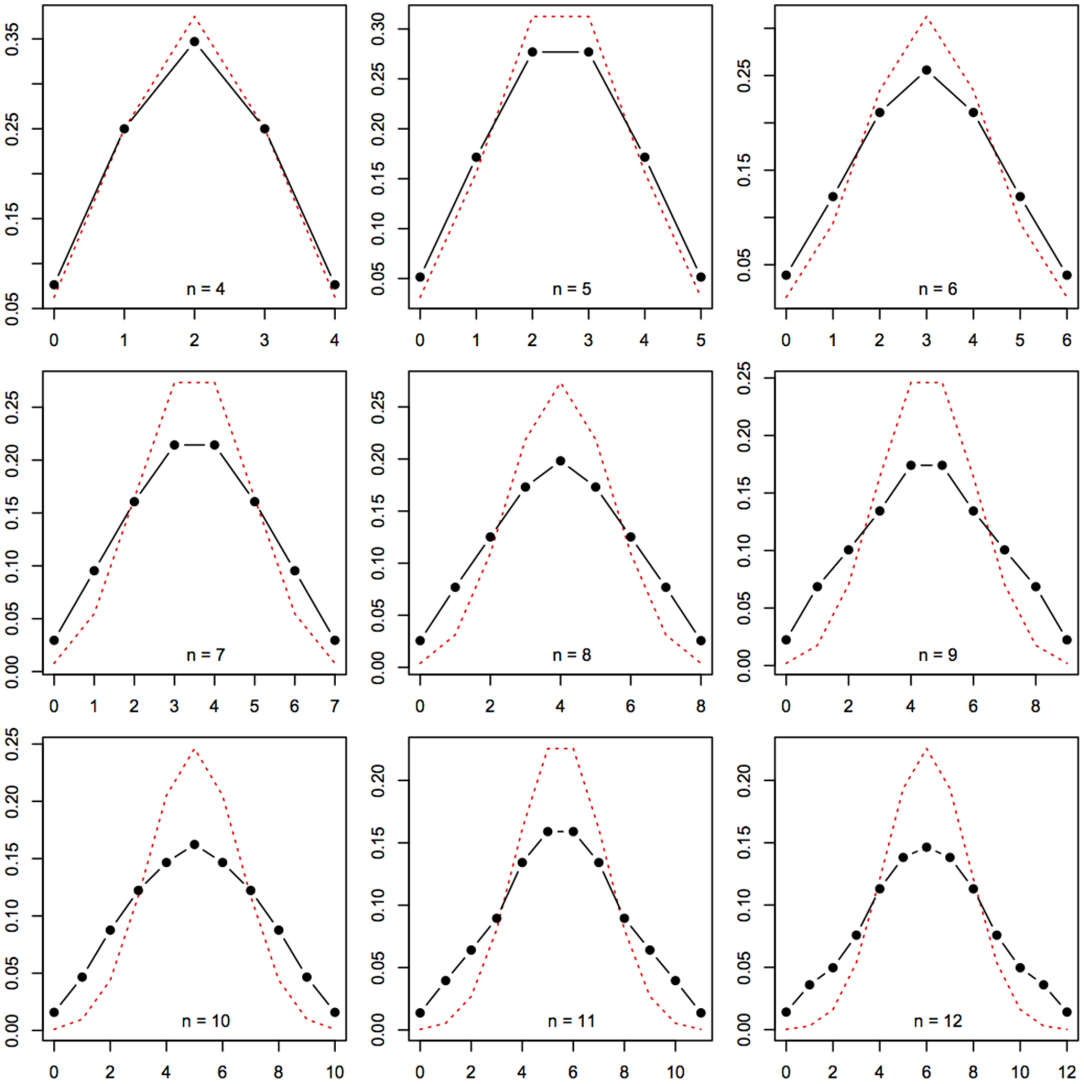
Distributions of the number of zeros in *n*-long binary sequences according to a truly random drawing (red, dotted), or a *D*(5) drawing (black, solid) for length 4 to 12.

In a random binary string of length 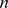, the number of ‘0s’ conforms to a binomial law given by 
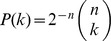
. On the other hand, if a random Turing machine is drawn, simpler patterns are more likely to appear. Therefore, the distribution arising from Turing machine should be more scattered, since most simple patterns are often unbalanced (such as 0000000). This is indeed what Fig. 7 shows: compared to truly random sequences of length 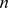, 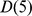 yields a larger standard deviation.

#### Bayesian application


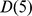
 allows us to determine, using a Bayesian approach, the probability that a given sequence is random: Let 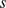 be a sequence of length 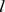. This sequence may be produced by a machine, let's say a 5-state Turing machine (event 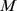), or by a random process (event 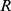). Let's set the prior probability at 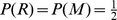. Because 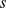 does not have a fixed length, we cannot use the usual probability 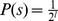, but we may, following Levin's idea, use 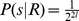. Given 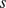, we can compute 
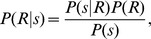
with 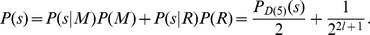



Since 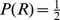 and 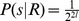, the formula becomes 
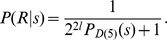



There are 16 strings 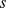 such that 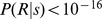 (the “least random strings”). Their lengths lie in the range 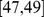. An example is: 11101110111011101110111011101110111011111010101. The fact that the “least random” strings are long can intuitively be deemed correct: a sequence must be long before we can be certain it is not random. A simple sequence such as 00000000000000000000 (twenty ‘0s’) gives a 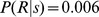.

A total of 192 strings achieve a 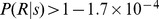. They all are of length 12 or 13. Examples are the strings 1110100001110, 1101110000110 or 1100101101000. This is consistent with our idea of a random sequence. However, the fact that only lengths 12 and 13 appear here may be due to the specificity of 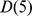.

#### Comparing *D*(4) and *D*(5)

Every 4-state Turing machine may be modeled by a 5-state Turing machine whose fifth state is never attained. Therefore, the 1832 strings produced by 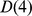 calculated in [13] also appear in 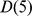. We thus have 1832 ranked elements in 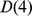 to compare with. The basic idea at the root of this work is that 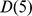 is a refinement (and major extension) of 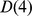, previously calculated in an attempt to understand and evaluate algorithmic complexity. This would be hopeless if 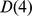 and 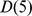 led to totally different measures and rankings of simplicity versus complexity (randomness).

#### Agreement in probability

**Figure 8:**
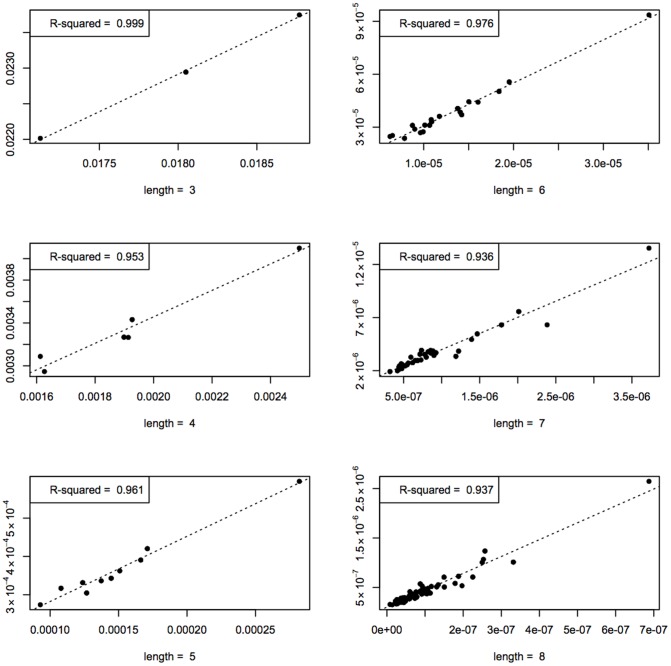
*D*(5) against *D*(4), for *n*-long strings.

The link between 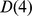 and 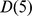 seen as measures of simplicity may be measured by the determination coefficient 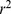, 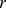 being the Pearson correlation coefficient. This coefficient is 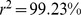, which may be understood as “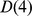 explains 99.23% of the variations of 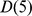
”. The scatterplot in Fig. 8 displays 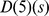 against 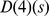 for all strings 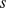 of length 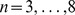 (8 being the largest integer 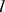 such that 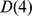 comprises every 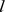-long sequence).

The agreement between 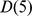 and 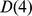 is almost perfect, but there are still some differences. Possible outliers may be found using a studentized residual in the linear regression of 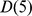 against 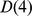. The only strings giving absolute studentized residuals above 20 are 0 and 1. The only strings giving absolute studentized residuals lying between 5 and 20 are all the 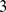-long strings. All 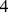-long strings fall between 2 and 5. This shows that the differences between 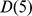 and 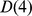 may be explained by the relative importance given to the diverse lengths, as shown above (Fig. 5).

#### Agreement in rank

**Figure 9:**
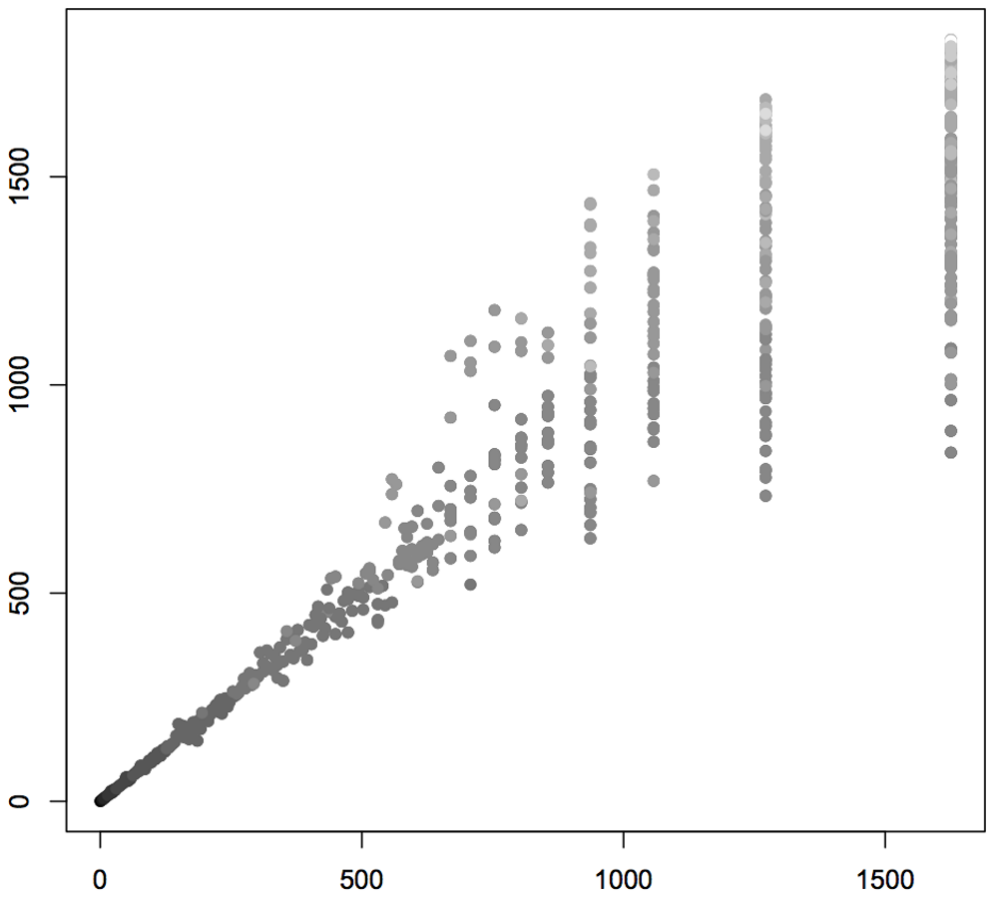
*R*_5_ (rank according to *D*(5)) against *R*_4_. The grayscale indicates the length of the strings: the darker the point, the shorter the string.

There are some discrepancies between 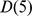 and 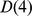 due to length effects. Another way of studying the relationship between the two measures is to turn our attention to ranks arising from 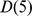 and 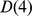. The Spearman coefficient is an efficient tool for comparing ranks. Each string may be associated with a rank according to decreasing values of 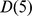 (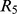) or 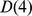 (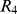). A greater rank means that the string is less probable. Fig. 9 displays a scatterplot of ranks according to 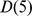 as a function of 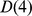-rank. Visual inspection shows that the ranks are similar, especially for shorter sequences. The Spearman correlation coefficient amounts to 0.9305, indicating strong agreement.

Not all strings are equally ranked and it may be interesting to take a closer look at outliers. Table 5 shows the 20 strings for which 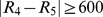. All these sequences are ties in 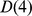, whereas 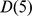 distinguishes 5 groups. Each group is made up of 4 equivalent strings formed from simple transformations (reversing and complementation). This confirms that 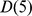 is fine-grained compared to 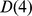.

The shortest sequences such that 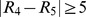 are of length 6. Some of them show an intriguing pattern, with an inversion in ranks, such as 000100 (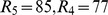) and 101001 with reversed ranks.

On the whole, 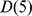 and 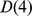 are similar measures of simplicity, both from a measurement point of view and a ranking point of view. Some differences may arise from the fact that 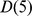 is more fine-grained than 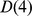. Other unexpected discrepancies still remain: we must be aware that 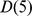 and 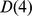 are both approximations of a more general limit measure of simplicity versus randomness. Differences are inevitable, but the discrepancies are rare enough to allow us to hope that 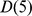 is for the most part a good approximation of this properties.

#### Kolmogorov complexity approximation

It is now straightforward to apply the Coding theorem to convert string frequency (as a numerical approximation of algorithmic probability 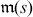) to estimate an evaluation of Kolmogorov complexity (see Tables 6 and 7) for the Turing machine formalism chosen. Based on the concept of algorithmic probability we define: 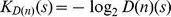



First it is worth noting that the calculated complexity values in Tables 6 and 7 are real numbers, when they are supposed to be the lengths of programs (in bits) that produce the strings, hence integers. The obvious thing to do is to round the values to the next closest integer, but this would have a negative impact as the decimal expansion provides a finer classification. Hence the finer structure of the classification is favored over the exact interpretation of the values as lengths of computer programs. It is also worth mentioning that the lengths of the strings (as shown in Table 7) are almost always smaller than their Kolmogorov (program-size) values, which is somehow to be expected from this approach. Consider the single bit. It not only encodes itself, but the length of the string (1 bit) as well, because it is produced by a Turing machine that has reached the halting state and produced this output upon halting.

Also worth noting is the fact that the strings 00, 01, 10 and 11 all have the same complexity, according to our calculations (this is the case from 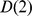 to 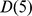). It might just be the case that the strings are too short to really have different complexities, and that a Turing machine that can produce one or the other is of exactly the same length. To us the string 00 may look more simple than 01, but we do not have many arguments to validate this intuition for such short strings, and it may be an indication that such intuition is misguided (think in natural language, if spelled out in words, 00 does not seem to have a much shorter description than the shortest description of 01).

Compare this phenomenon of program-sizes being greater than the length of these short strings to the extent of the problem posed by compression algorithms, which collapse all different strings of up to some length (usually around 100 bits), retrieving the same complexity approximation for all of them despite their differences. One way to overcome this minor inconvenience involved in using the alternative approach developed here is to subtract a constant (no greater than the smallest complexity value) from all the complexity values, which gives these strings lower absolute random complexity values (preserving the relative order). But even if left “random”, this alternative technique can be used to distinguish and compare them, unlike the lossless compression approach that is unable to further compress short strings.

The phenomenon of complexity values greater than the lengths of the strings is transitional. Out of the 99,608 strings in 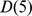, 212 have greater string lengths than program-size values. The first string to have a smaller program-size value than string length is the string 10101010101010101010 101010101010101010101 (and its complementation), of length 41 but program-size of 33.11 (34 if rounded). The mean of the strings with greater program-size than length is 38.35, The string with the greatest difference between length and program-size in 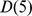 are strings of low Kolmogorov complexity such as 0101010001000100010001000100010001000100010001010, of length 49 but with an approximated Kolmogorov complexity (program-size) value of 39.06. Hence far from random, both in terms of the measure and in terms of the string's appearance.

#### Randomness in *D*(5)

Paradoxically, the strings at the bottom of 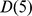 as sorted from highest to lowest frequency and therefore lowest to highest Kolmogorov (random) complexity are not very random looking, but this is to be expected, as the actual most random strings of these lengths would have had very low frequencies and would not therefore have been produced. In fact what we are looking at the bottom are some of the longest strings with greatest structure (and hence with lowest Kolmogorov complexity) in 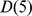. Table 8, however, shows the bottom of the length 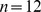 classification extracted from 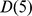, for which all, but 2, 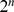 binary strings were produced, hence displaying more apparent randomness in the subset of length 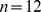 but still likely unstable due to the fact that they were produced by a relatively small number of Turing machines (in fact 2 strings of length 12 were never produced), compared to the ones at the top for 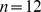 that, as we have shown, can be expected to be stable (from comparing 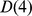 and 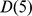).

#### Robustness of 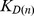


An important question is how robust is 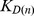, that is how sensitive it is to 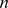. We know that the invariance theorem guarantees that the values converge in the long term, but the invariance theorem tells nothing about the rate of convergence. We have shown that 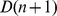 respects the order of 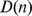 except for very few and minor value discrepancies concerning the least frequent strings (and therefore the most unstable given the few machines generating them). This is not obvious despite the fact that all Turing machines with 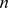 states in 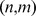 are included in the space of 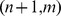 machines (that is, the machines that never reach one of the 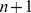 states), because the number of machines in 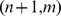 overcomes by far the number of machines in 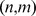, and a completely different result could have been then produced. However, the agreement between 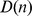 and 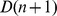 seems to be similarly high among, and despite, the few cases 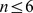 in hand to compare with. The only way for this behaviour to radically change for 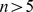 is if for some 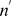, 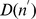 starts diverging in ranks from 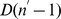 on before starting to converge again (by the invariance theorem). If one does not have any reason to believe in such a change of behavior, the rate of rank convergence of 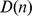 is close to optimal very soon, even for the relatively “small” sets of Turing machines for small 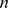.

One may ask how robust the complexity values and classifications may be in the face of changes in computational formalism (e.g. Turing machines with several tapes, and all possible variations). We have shown [28] that radical changes to the computing model produce reasonable (and correlated with different degrees of confidence) ranking distributions of complexity values (using even completely different computing models, such as unidimensional deterministic cellular automata and Post tag systems).

We have also calculated the maximum differences between the Kolmogorov complexity evaluations of the strings occurring in every 2 distributions 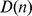 and 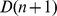 for 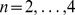. This provides estimations for the constant 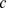 in the invariance theorem (Eq. 2) determining the maximum difference in bits among all the strings evaluated with one or another distribution, hence shedding light on the robustness of the evaluations under this procedure. The smaller the values of 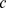 the more stable our method. The values of these bounding constants (in bits) among the different numerical evaluations of 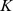 using 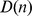 for 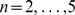 after application of the Coding theorem (Eq. 3) are: 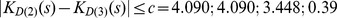


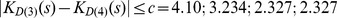


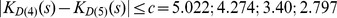



Where 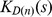 means 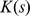 evaluated using the output frequency distribution 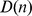 after application of the Coding theorem (Eq. 3) for 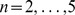 (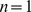 is a trivial non interesting case) and where every value of 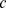 is calculated by quartiles (separated by semicolons), that is, the calculation of 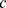 among all the strings in the 2 compared distributions, then among the top 3/4, then the top half and finally the top quarter by rank. Notice that the estimation of 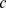 between 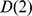 and 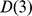, and 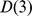 and 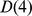 remained almost the same among all strings occurring in both, at about 4 bits. This means one could write a “compiler” (or translator) among the two distributions for all their occurring strings of size only 4 bits providing one or the other complexity value for 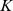 based on one or the other distribution. The differences are considerably smaller for more stable strings (towards the top of the distributions). One may think that given that the strings with their occurrences in 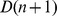 necessarily contain those in 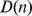 for all 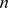 (because the space of all Turing machines with an additional state always contain the computations of the Turing machines will less states), the agreement should be expected. However, the contribution of 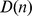 to 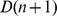 contributes with 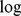 the number of strings in 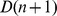. For example, 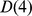 contributes only 1832 strings to the 99 608 produced in 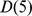 (that is less than 2%). All in all, the largest difference found between 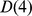 and 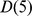 is only of 5 bits of among all the strings occurring both in 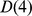 and 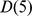 (1832 strings), where the values of 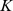 in 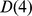 are between 2.285 and 29.9.

## Concluding remarks

We have put forward a method based on algorithmic probability that produces frequency distributions based on the production of strings using a standard (Rado's) model of Turing machines generally used for the Busy Beaver problem. The distributions show very small variations, being the result of an operation that makes incremental changes based on a very large number of calculations having as consequence the production of stable numerical approximations of Kolmogorov complexity for short strings for which error estimations of 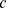 from the invariance theorem were also estimated. Any substantial improvement on 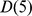, for example, by approximation of a 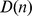 for 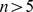, is unlikely to happen with the current technology as the number of Turing machines grows exponentially in the number of states 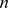. However, we have shown here based both on theoretical and experimental grounds that one can choose informed runtimes significantly smaller than that of the Busy Beaver bound and capture most of the output determining the output frequency distribution. An increase of computational power by, say, one order of magnitude will only deliver a linear improvement on 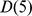.

The experimental method presented is computationally expensive, but it does not need to be executed more but once for a set of (short) strings. As a result this can now be considered an alternative to lossless compression as a complementary technique for approximating Kolmogorov complexity. An *Online Algorithmic Complexity Calculator* (OACC) implementing this technique and releasing the data for public use has been made available at http://www.complexitycalculator.com.

The data produced for this paper has already been used in connection to graph theory and complex networks [30], showing, for example, that it produces better approximations of Kolmogorov complexity of small graphs (by comparing it to their duals) than lossless compressibility. In [25] it is also shown how the method can be used to classify images and space-time diagrams of dynamical systems, where its results are also compared to the approximations obtained using compression algorithms, with which they show spectacular agreement. In [33], it is used to investigate the ratios of complexity in rule spaces of cellular automata of increasing size, supported by results from block entropy and lossless compressibility. In [15], it is also used as a tool to assess subjective randomness in the context of psychometrics. Finally in [23], the method is used in numerical approximations to another seminal measure of complexity (Bennett's Logical Depth), where it is also shown to be compatible with a calculation of strict (integer-value) program-size complexity as measured by an alternative means (i.e. other than compression). The procedure promises to be a sound alternative, bringing theory and practice into alignment and constituting evidence that confirms the possible real-world applicability of Levin's distribution and Solomonoff's universal induction (hence validating the theory itself, which has been subject to criticism largely on grounds of simplicity bias and inapplicability). As Gregory Chaitin has pointed out [7] when commenting on this very method of ours:

The theory of algorithmic complexity is of course now widely accepted, but was initially rejected by many because of the fact that algorithmic complexity depends on the choice of universal Turing machine and short binary sequences cannot be usefully discussed from the perspective of algorithmic complexity. … discovered employing [t]his empirical, experimental approach, the fact that most reasonable choices of formalisms for describing short sequences of bits give consistent measures of algorithmic complexity! So the dreaded theoretical hole in the foundations of algorithmic complexity turns out, in practice, not to be as serious as was previously assumed. … [hence, of this approach] constituting a marked turn in the field of algorithmic complexity from deep theory to practical applications.

This also refers to the fact that we have found an important agreement in distribution–and therefore of estimations of Kolmogorov complexity upon application of the algorithmic Coding theorem–with other abstract computing formalisms such as one-dimensional cellular automata and Post's tag systems [62]. In this paper we have provided strong evidence that the estimation and scaling (albeit limited by computational power) of the method is robust and much less dependent on formalism and size sample than what originally could have been anticipated by the invariance theorem.

## Materials and Methods

Additional material can be found at the *Algorithmic Nature Group* website (http://www.algorithmicnature.org). An Online Algorithmic Complexity Calculator implementing this technique and making the data available to the research community is accessible at http://www.complexitycalculator.com.
